# Real-world questions and concerns about disease-modifying antirheumatic drugs (DMARDs): a retrospective analysis of questions to a medicine call center

**DOI:** 10.1186/s41927-020-00126-7

**Published:** 2020-06-16

**Authors:** Hiba EL Masri, Samantha A. Hollingworth, Mieke van Driel, Helen Benham, Treasure M. McGuire

**Affiliations:** 1 School of Pharmacy, The University of Queensland, 20 Cornwall St, Woolloongabba, QLD 4102 Australia; 2 Primary Care Clinical Unit, Faculty of Medicine, The University of Queensland, Brisbane, Australia; 3 Faculty of Medicine, The University of Queensland, Brisbane, Queensland Australia; 4 Department of Rheumatology, Princess Alexandra Hospital, Brisbane, QLD 4102 Australia; 5 Faculty of Health Sciences and Medicine, Bond University, Robina, QLD 4226 Australia; 6 Mater Pharmacy, Mater Health, Raymond Tce, South Brisbane, QLD 4101 Australia

**Keywords:** Disease-modifying antirheumatic drugs, Information services, Patients, Help-seeking behavior OR information-seeking behaviour, Drug information services, Patient perspective, Concern, Expected outcome

## Abstract

**Background:**

Disease-modifying antirheumatic drugs (DMARDs) have transformed the treatment of numerous autoimmune and inflammatory diseases but their perceived risk of harm may be a barrier to use.

**Methods:**

In a retrospective mixed-methods study, we analysed conventional (c) and biologic (b) DMARDs-related calls and compared them with rest of calls (ROC) from consumers to an Australian national medicine call center operated by clinical pharmacists from September 2002 to June 2010. This includes the period where bDMARDs became available on the Pharmaceutical Benefits Scheme, the government-subsidized prescription medicines formulary. We compared caller and patient demographics, enquiry types and motivation to information-seek for both cDMARDs and bDMARDs with ROC, using a t-test for continuous data and a chi-square test for categorical data. We explored call narratives to identify common themes.

**Results:**

There were 1547 calls involving at least one DMARD. The top three cDMARD enquiry types were side effects (27.2%), interactions (21.9%), and risk versus benefit (11.7%). For bDMARDs, the most common queries involved availability and subsidized access (18%), mechanism and profile (15.8%), and side effects (15.1%). The main consumer motivations to information-seek were largely independent of medicines type and included: inadequate information (44%), wanting a second opinion (23.6%), concern about a worrying symptom (18.8%), conflicting information (6.9%), or information overload (2.3%). Question themes common to conventional and biological DMARDs were caller overemphasis on medication risk and the need for reassurance. Callers seeking information about bDMARDs generally overestimated effectiveness and focused their attention on availability, cost, storage, and medicine handling.

**Conclusion:**

Consumers have considerable uncertainty regarding DMARDs and may overemphasise risk. Patients cautiously assess the benefits and risks of their DMARDs but when new treatments emerge, they tend to overestimate their effectiveness.

## Background

The clinical trial evidence for disease modifying antirheumatic drugs (DMARDs) to treat various autoimmune diseases emerged in the 1980s for conventional (cDMARDs) and in the 1990s for biologic (bDMARDs). The latter only became widely accessible to the Australian public with listing on the Pharmaceutical Benefits Scheme for rheumatoid arthritis (RA) in 2003 [1]. DMARDs have revolutionized the management of conditions such as RA and other autoimmune diseases [2–4]. Although a growing body of evidence demonstrates that their use is relatively safe [5], consumers report information gaps and express concerns related to the perceived aggressive and harmful nature of DMARDs [6]. Patients on DMARDs have complex and often evolving perceptions about their long-term side effects and efficacy [7–10]. Concerns and beliefs about DMARDs can affect adherence to treatment and eventually clinical outcomes [11, 12]. Stronger beliefs in the necessity of DMARDs are associated with better adherence, while increased concerns about their safety correlates with poorer adherence [13, 14]. Sociodemographic variables, including age, education, ethnicity, and income are also found to impact patients’ perceptions about DMARDs. Younger, more educated, and patients with higher income have a greater risk tolerance [15] whereas black patients are found to be more risk averse than non-black patients [16].

Although previous studies have enabled a partial understanding of patients’ perspectives on DMARDs, we know little about the information gaps and concerns of Australian consumers, particularly as clinician prescribing has evolved from the use of only cDMARDs to include bDMARDs [15, 16]. This study therefore aims to characterise real-world questions and concerns about DMARDs, both conventional and biologic, through the analysis of calls made to a national medicine call center (MCC) in Australia over an eight-year timeframe, inclusive of the period where bDMARDs became available on the Pharmaceutical Benefits Scheme (PBS) which provides government-subsidized prescription medicines to citizens and permanent residents.

## Methods

### Data collection and management

A retrospective, mixed methods analysis of consumers’ enquiries to a national consumer MCC about the use of both conventional and biologic DMARDs was undertaken. Calls were extracted with questions relating to DMARDs from NPS MedicineWise (formerly National Prescribing Service (NPS) *Medicines Line*), operated by clinical pharmacists at Mater Health, Brisbane between September 2002 and June 2010. The service was funded by NPS to serve consumers throughout Australia for the cost of a local call. As data were originally collected for routine health service without specific a priori research goals, this research followed the REporting of studies Conducted using Observational Routinely-collected health Data (RECORD) guideline [17], an extension of the STrengthening the Reporting of OBservational studies in Epidemiology (STROBE) guidelines [18].

The details of each call were captured on a standardised form and entered into a Microsoft Access database. These included demographic characteristics of both patient and caller, enquiry type, relationship of caller to patient, postcode, and motivation to call. For each call, up to three generic medicines relating to the question were recorded and categorised by the Anatomical Therapeutic Chemical (ATC) classification of medicines [19]. The pharmacists also noted on the forms the questions asked, using the same terms and wordings of the consumers. We extracted all calls related to DMARDs (L04AX for methotrexate and leflunomide, M01CB for auranofin, A07EC for sulfasalazine, P01BA for chloroquine and hydroxychloroquine, and L04AB for all the bDMARDs). The remaining calls were classified as ‘rest of calls’ (ROC).

### Quantitative analysis

DMARD-related calls were analyzed and compared with ROC over an eight-year period. Caller and patient demographic characteristics, enquiry types and caller motivations to help-seek were compared between DMARD (conventional, biologic, and combined) calls and ROC using a t-test for continuous data and a chi-square test for categorical data. Any difference with a two-sided *p*-value < 0.05 was considered statistically significant. When testing statistical significance versus ROC, *p* values were adjusted using Bonferroni correction to minimize the risk of type I errors [20]. Caller postcodes from each cohort were mapped to the Accessibility/Remoteness Index of Australia (ARIA) which determines the remoteness of areas from service centres [21, 22]. The proportion of calls from each ARIA were compared with Australian Bureau of Statistics population data to obtain relative call frequencies expressed as a ratio [21, 22]. We analyzed the quantitative data using IBM SPSS Statistics 25 [23].

### Narrative analysis

The narratives associated with call questions that were available electronically were thematically analyzed and coded through an interactive and iterative process undertaken by two investigators independently (TM and HM) [24]. Codes were compared, discussed, and collapsed into major and minor themes, and any associated sub-themes. A theme was considered major if it constituted 10% or more of all DMARD narratives. A theme emerging from at least 1% of all narratives (and less than 10%) was considered a minor theme; while themes for less than 1% of narratives were not considered in the results. For major themes, we captured any substantive differences in the profile between cDMARDs and bDMARDs.

## Results

### Demographics and quantitative analysis

Of the 123,217 calls to the MCC, 1547 involved at least one DMARD, with half (50.6%) including methotrexate alone or in combination with a bDMARD. Hydroxychloroquine, sulfasalazine, and leflunomide were the next most common cDMARDs (14.6, 14.5, and 7.8%). One in five enquiries concerned a bDMARD including etanercept, adalimumab, infliximab, and rituximab (7.4, 5.4, 3.2, and 3.1%).

DMARD callers, as well as patients, were predominantly female (82.4 and 78.1%), with most calls (79%) received from patients themselves (Table 1). The mean age of callers was older for DMARDs than for ROC (53.63 ± 17.76 vs. 46.82 ± 23.785 years). There was a significantly higher proportion of partners and family members calling to enquire specifically about a bDMARD than for ROC (28.9% vs. 14.5%). DMARD callers hailed from all Australian states and territories and across metropolitan, rural and remote areas (Table 1).

**Table 1:** Demographics and characteristics of national MCC calls, DMARD-related versus ROC

Characteristic	All DMARDs (***n*** = 1547)	cDMARDs (***n*** = 1269)	bDMARDs (***n*** = 278)	ROC (***n*** = 121,709)
Gender of the caller (%)				
Female	82.4†	83.8†	74.9‡	76.7
Male	17.6†	16.2†	25.1‡	23.1
Missing	–	–	–	0.2
Gender of the patient (%)				
Female	78.1†	80.4†	65.6†‡	68.3
Male	21.8†	19.5†	34.3†‡	31.4
Missing	0.1	< 0.1	< 0.1	0.3
Age of the patient	53.63†	53.62†	52.74†	46.82
Mean (± SD) years	(±17.76)	(±17.89)	(±17.44)	(±23.75)
Missing (%)	0.45%	0.39%	0.36%	–
Relationship of the caller (%)				
Self	79.0†	81.2†	67.8‡	71.3
Partner	7.2†	6.5†	10.9†‡	1.7
Child	5.7†	5.4†	7.5†‡	2.6
Other family or friend	5.6†	4.7†	10.5‡	10.2
Other	2.5	2.5	3.3†	1.9
ARIA relative call frequency				
Highly accessible	1.23	1.23	1.20	1.24
Accessible	0.50	0.50	0.55	0.46
Moderately accessible	0.39	0.39	0.38	0.32
Remote	0.34	0.34	0.96†‡	0.69
Very remote	0.44	0.59	0	0.73
Missing (% of calls)	2.5	2.4	2.9	2.7

*ARIA* The Accessibility/Remoteness Index of Australia

† *p < 0.05* versus ROC, with Bonferroni correction

‡ *p < 0.05* versus cDMARDs

The top three enquiry types for all DMARD calls involved questions about medicines safety: side-effects (25.2%), interactions (19.4%), and risk/benefit assessment and comparison among DMARDs (12.1%), all significantly higher than in ROC (Table 2). Other frequent enquiries for both DMARDs and ROC included mechanism or profile of action of the medicine, as well as the indication or use as a treatment option. There was a different ranking of key enquiry types for bDMARDs alone excluding the most common: enquiries on side effects (15.1%). Calls for bDMARDs frequently focused on consumers wanting to understand how these medicines worked to effectively treat various conditions: mechanism/profile of action (15.8%), indication and treatment (10.8%), efficacy (5.8%), or to enquire about logistical issues such as availability (11.2%), cost and subsidized access (8.2%), identification and/or formulation (4.7%), and stability, storage, and disposal (4.7%). The major consumer motivations to information-seek regarding DMARDs were largely independent of medicines type (Table 2) and were prompted by inadequate information (44%), needing a second opinion (23.6%), concern about a worrying symptom (18.8%), conflicting information (6.9%), or information overload (2.3%). Many callers were driven to help-seek due to inadequate information about bDMARD (57.7% of calls).

**Table 2:** Enquiry types and motivations to call for DMARDs versus ROC

	All DMARDs (***n*** = 1547) (%)	cDMARD (***n*** = 1269) (%)	bDMARDs (***n*** = 278) (%)	ROC (***n*** = 121,709) (%)
**Enquiry type**				
Side-effects	25.2†	27.2†	15.1†‡	19.6
Interaction	19.4†	21.9†	6.5†‡	15.1
Risk/Benefit & comparison	12.1†	12.5†	9.7‡	8.3
Mechanism / Profile	9.5†	8.4	15.8†‡	7.0
Indication & treatment	10.5	10.5	10.8	11.9
Dose & administration	6.0	6.7	2.5†‡	7.9
Efficacy	3.1	2.8	5.8†‡	2.8
Availability	3.0	1.4	11.2†‡	2.6
Pregnancy & lactation	2.9†	3.1†	1.8†	8.6
Cost and PBS access	2.0	0.7	8.2†‡	1.4
Identification / Formulation	1.9	1.2	4.7‡	3.4
Stability/Storage/Disposal	1.3	0.6	4.7†‡	0.9
Abuse and withdrawal	1.4	1.5	0.4†	3.4
Generics	0.4	0.3	0.7	0.8
Vaccination	0.7	0.7	0.7	1.9
Outside service brief (e.g.	0.1	0.1	0	1.5
poisons query)				
Non-medicines issue	0.6	0.5	1.1	2.
**Motivation to call**				
Inadequate information	44.0	44.0	57.7†‡	46.6
Second opinion	23.6	23.6	18.4†‡	23.5
Worrying symptom	18.8	18.8	13.4†‡	17.8
Conflicting information	6.9†	6.9†	1.7†‡	~ 0
Information overload	2.3	2.3	1.7	1.2
CMI	0.9	0.9	0.1	0.5
Forgot information	0.7	0.7	1.3	0.1
Media	1.2	1.2	3.4†‡	0.9
Health professionals	0.5	0.5	0.8	6.3
Other	1.1	1.1	1.7	1.7

*PBS* Pharmaceutical Benefits Scheme, *CMI* Consumer Medicines Information

† *p < 0.05* versus ROC, with Bonferroni correction

‡ *p < 0.05* versus cDMARDs

### Narrative analysis

There were 724 call narratives that included a DMARD, but 43 narratives contained questions that focused on other medicine(s) used or of interest to the patient and were excluded from the analysis. Thematic analysis of the remaining 681 narratives revealed four major themes, each including several sub-themes, and four minor themes (Table 3).

**Table 3:** Themes emerging from the narrative analysis of DMARDs calls

Themes	All DMARDs % (***n*** = 681)	cDMARDs % (***n*** = 591) (%)	bDMARDs % (***n***=90) (%)
**Major themes & subthemes**			
**1. Concern about medication safety**	26.9	28.4	20.8
1.1. Clarification on potential side effect of a specified DMARD *“Will methotrexate cause hair loss?”*	20.2	21.0	16.8
1.2. Information on the general safety of a specified DMARD *“What are the side effects of adalimumab?”*	4.0	4.3	2.4
1.3. Reassurance on the use of a specified DMARD *“What are the risks of taking methotrexate as I am worried about taking it after the things I read on the internet?”*	2.7	3.1	1.6
**2. Seeking information on potential interactions with a specified DMARD**	22.4	25.6	8.0
2.1. Seeking information on potential interactions of medicines with a specified DMARD *“Can hydroxychloroquine be taken with diclofenac?”*	16.9	19.2	6.4
2.2. Seeking information on potential interactions of complementary medicines with a specified DMARD *“Can I take St john’s wort if I am on Mabthera* (rituximab)*?”*	3.5	4.1	0.8
2.3. Lifestyle while on a specified DMARD *“Would there be an interaction between methotrexate and some alcohol I had at Christmas?”*	1.6	1.9	0.0
2.4. Seeking information on potential interactions of a specified DMARD with food *“Any interactions between food and Humira (adalimumab) injection?”*	0.4	0.4	0.8
**3. Seeking a therapeutic strategy**	13.0	14.7	4.0
3.1. Seeking a therapeutic strategy regarding the timing, dose, and administration of a specified DMARD *“Do I inject methotrexate intramuscularly or subcutaneously?”*	7.5	8.0	3.2
3.2. Seeking a therapeutic strategy regarding dosing and timing of concomitant medicines to DMARD therapy *“What is the normal dose of folic acid when I am also on methotrexate 20 mg per week?”*	1.0	1.0	0.0
3.3. Seeking a therapeutic strategy for managing withdrawal and/or reintroduction of a specified DMARD *“Can he stop methotrexate suddenly or does he have to do so gradually?”*	2.6	3.2	0.0
3.4. Seeking a therapeutic strategy regarding the management of an adverse event and/or the continuation of DMARD therapy in case of an occurred adverse event *“What can I do if I’ve experienced hives and irritation since starting adalimumab?”*	1.5	1.6	0.8
3.5. Seeking a therapeutic strategy to manage a DMARD therapy before and/or after a procedure or an elective surgery *“When should I cease meloxicam and sulfasalazine before surgery?”*	0.7	0.9	0.0
**4. Seeking information on a specified DMARD**	10.1	4.9	33.6
4.1. Seeking general information on a specified DMARD *“Can you please provide me with information on hydroxychloroquine?”*	4.4	3.6	8.0
4.2. Seeking information on a specified DMARD in response to media *“Disabled friend in his 70s saw a TV documentary on a new drug for arthritis- what is it?”*	2.3	0.2	12.0
4.3. Seeking information on the availability, cost, and logistics to access a specified DMARD *“How can I obtain an emergency supply of Enbrel (etanercept) when my specialist is away?”*	1.9	1.1	5.6
4.4. Seeking information on the restrictions and requirements to obtain subsidized DMARD *“Why can’t my 16-year-old nephew get Humira (adalimumab) on the PBS?”*	1.5	0.2	8.0
**Minor themes**			
1. Quantifying the benefit and harm of a specified DMARD *“What are the risks verse benefits of taking methotrexate for the treatment of psoriasis?”*	7.5	7.5	7.2
2. Concern on the impact of a DMARD on reproductive health *“Could any of my partner’s medicines cause abnormalities in my child: prednisone, methotrexate, leflunomide?”*	4.3	4.7	2.4
3. Storage, handling and stability of a specified DMARD *“How do I know if Humira (adalimumab) is okay if it was left out of the fridge for over 24 h?”*	1.5	0	8.0
4. Concern about the safety of contact with a DMARD user *“Can my daughter have contact with pregnant women or children if she is on methotrexate?”*	1.0	1.3	0.2

Callers’ primary concerns about DMARDs, whether conventional or biological, centred on medication safety, accounting for more than a quarter (26.9%) of all narratives. Many callers sought clarification as to whether a symptom being experienced was a side effect caused by their DMARD. Among these narratives, there were questions such as *“Can methotrexate cause facial skin lesions which get scabby and burn?”* Other sub-themes involved callers asking about the safety of their DMARD in general; and those seeking reassurance on the use of their DMARD (Table 3).

Potential interactions with DMARDs (22.4%) were the second major consumer focus, with most queries concerning cDMARDs. Callers enquired about possible interactions between their DMARD (or one proposed for use) with their other medicines. These queries most commonly involved nonsteroidal anti-inflammatory drugs (NSAIDs), cardiovascular and central nervous system agents, vaccines, and complementary medicines. The likelihood of an interaction between a DMARD with food, alcohol, smoking, or recreational substances such as ecstasy was also raised.

In 13% of narratives, callers were seeking a DMARD-related therapeutic strategy. These themes primarily involved cDMARDs. Some calls referred to a relatively-immediate action regarding the timing, dose, or administration of a specified DMARD, while other queries focused on the dosing and timing of a concomitant medicine in relation to DMARD therapy (mostly folic acid and methotrexate). Although less frequent, there were key subthemes where callers requested a strategy to manage their DMARD in a range of circumstances: where the DMARD was to be withdrawn or reintroduced; whether the medicine should be withdrawn after an adverse event; and clarification of appropriate timing of the DMARD dose before or after a procedure, for example: *“Can I take my methotrexate dose today if I have just had a local anaesthetic at my dentist?”*

The final major theme involved callers seeking information on a specified DMARD. While these constituted only 10.1% of narratives, there was a strong question focus on bDMARDs. One third of all requests for further information concerning the then-novel bDMARDs were generated by discussion in the media. We detected two waves of calls following two distinct television reports, each discussing the introduction of a new bDMARD for the treatment of arthritis. Callers’ enquiries ranged from questions about this “*new injectable drug”* they *“heard about on TV news”* to statements such as *“they said it is a new wonder drug”.* Of note, very few callers sought to clarify *“is it for me?”* or *“is enough known?”* Related subthemes were callers seeking: general information about a specific DMARD; information on the availability, cost, and logistics to access a DMARD; and the restrictions and requirements to obtain a subsidized DMARD, for example: *“I’ve been on Remicade (infliximab) for the last 18 months, can I get it cheaper than $800?”*

Four minor but noteworthy themes also emerged from this narrative analysis. The first was where callers sought assistance to quantify the benefit versus harm of a specified DMARD (7.5%), conventional or biological. In some cases, this was to compare different DMARDs for a given clinical scenario: *“Which is the better treatment for rheumatoid arthritis: Enbrel (etanercept) or Humira (adalimumab)?”* or to probe for an alternative with better efficacy and or less side effects: *“Humira (adalimumab) – is it more effective or have less side effects?”*

Another issue of significant concern for callers was the potential impact of a DMARD on reproductive health (4.3%), with a general overestimation of risk. This theme included narratives about the safety of various DMARDs:

- ▪ In the period prior to conception, for example: “*How long after methotrexate can I try to become pregnant?*”
- ▪ For either gender at different time points during pregnancy: “*My partner has been taking methotrexate (25mg per week) and I have fallen pregnant. Will the baby be affected*?”
- ▪ During breastfeeding: “*Would Plaquenil (hydroxychloroquine) be likely to cause my five-week old breastfed baby to still be jaundiced?”* and *“Can I continue breastfeeding my baby if I have been prescribed sulfasalazine and prednisolone for rheumatoid arthritis?*”
- ▪ In relation to environmental exposure: “*Am I going to be a concern to my pregnant daughter because I am taking weekly methotrexate?”* Callers were also anxious about the negative impact of a DMARD on male fertility: “*What is the effect of taking methotrexate on sperm?*”

Storage, handling and stability of a specified DMARD constituted a minor theme (1.5% DMARDs and 8% bDMARDs). Callers wanted information about the stability of a biologic after removing it from the fridge or after freezing and defrosting it, for example: *“Can I still use Humira* (adalimumab) *if it was completely frozen for twelve hours but has since defrosted and looks perfectly normal?”* Some callers were also looking for solutions to keep their medicine at an appropriate temperature while traveling: *“How is best to store my etanercept if I am going overseas?”*

Finally, some callers were concerned about the safety of coming in contact with a DMARD user, specifically children and immunocompromised persons. This theme included questions such as *“Do I need to stay away from my grandchild after being administered low-dose methotrexate?”* and “*If I take Methoblastin (methotrexate) will I be unable to touch my animals or family and friends?”*

## Discussion

The calls to the MCC demonstrate that consumers have many unanswered questions and considerable uncertainty regarding their DMARDs. Uncertainty is acknowledged as an integral component of health and illness [25]. It manifests when situations are ambiguous, complex or unpredictable; when information is inadequate or inconsistent; and when people feel insecure in their own state of knowledge or the state of knowledge in general [26]. A key strategy to cope or manage uncertainty is information seeking [25, 27, 28]. This is consistent with our study findings where DMARD information seeking was motivated by callers needing a second opinion, being concerned about a worrying symptom and/or having inadequate or conflicting information. This often occurs in chronic disease where more information sources may be accessed and there is a greater opportunity to encounter inconsistent information [29]. This may potentially cause increased patient anxiety [30] and altered risk perception [31]. Conflicting information can also impact negatively on adherence and cause patients to alter their medication regimen without consulting their physician [32].

In addition to information seeking, callers requested immediate therapeutic strategies from a trusted source to manage various situations including dosing of DMARDs and concomitant medicines, withdrawal, management of an adverse event, and the interruption of medication for surgical procedures. This suggests that it can be a challenge for consumers to access reliable DMARD information; this poor access may be perceived as a barrier to meeting their health information needs and achieving their therapeutic goals [33].

In Australia, patients with a chronic disease often need to travel large distances to visit a doctor. In our study, DMARD-related callers emanated from every state and territory, including rural and remote areas. The MCC provided an accessible alternative for people who were seeking anonymity but required timely medicines information from a trusted source [34].

Most enquiries were generated by female callers. This is consistent with health information seeking literature, where women generally seek medicines information more often than males [35, 36]. The majority of patients were also female which reflects the general nature of autoimmune diseases treated by DMARDs [37]. There were more calls from the patients themselves regarding DMARDs than in ROC, which potentially reflects ownership of their condition and higher involvement in management, a trait consistent across many chronic diseases such as RA [38, 39].

Most consumers were motivated to proactively information-seek due to perceived risks of DMARDs, with many seeking assistance from the MCC pharmacist to assess the risks and benefits of their DMARD. Callers sought clarification about potential side effects and a therapeutic strategy to manage DMARD use. Patients with inflammatory arthropathies in various populations often overestimate the risk of side effects, particularly with long-term treatment [6, 8, 40, 41]. Safety as a goal of medicines information seeking is supported by consumer medicines information needs literature [42]. High fear scores can reduce adherence and potentially impede clinical outcomes [43–45]. In our DMARD cohort, many callers expressing their concern of taking the medicine resulted from reading the consumer package insert. Since 1993, Australian legislation requires all new prescription and pharmacist-only medicines to be accompanied by a package insert, Consumer Medicines Information (CMI), prepared by the sponsor. These aim to provide consumers with accurate, unbiased, and easy to understand medicines information for informed decision-making [46]. While CMI layout is guided by legislation, it does not mandate quality and consistency of content [47]. There is no requirement for the sponsor to routinely update CMI to reflect information currency or adequately address risk in special populations. These factors may contribute to consumer confusion and in some cases cause fear [48]. Current CMI for various bDMARDs in Australia, also available to the public on the Australian Therapeutic Goods Administration website, report on the frequency of serious undesirable effects, particularly serious infections and malignancies, based on their original clinical trials [49]. However, over the last 20 years evidence from larger registry populations using bDMARDs have demonstrated the safety of these medicines [5]. This relatively high safety profile is not reflected in the official material that patients may read. The situation is similar for the Summary of Product Characteristics used in Europe and the Package Label used in the United States and may contribute to the public’s overestimation of risk with this medicines class.

A key concern raised by consumers was DMARD drug interactions where the major medicines were another DMARD, NSAIDs, cardiovascular and central nervous system agents, vaccines, and complementary medicines. The concomitant use of these medicines in inflammatory arthropathies is common. Studies have not previously identified this consumer concern [50–53].

In addition to caller focus on potential DMARD side-effects and interactions, we observed the impact of the media during the emergence of bDMARD availability in Australia. These calls indicated consumer overestimation of effectiveness. Hoffman and Del Mar conducted a comprehensive systematic review of studies that quantitatively assessed patients’ expectations of the benefits and/or harms of any treatment, test, or screening test [52]. They found participants rarely had accurate expectations of benefits and harms, and for many interventions, they had a tendency to overestimate benefits and underestimate harms. For the two studies that measured medicine expectations for the bDMARD infliximab in inflammatory bowel disease [54, 55], at least half of participants overestimated benefit in eight of ten outcomes. The authors suggested that there appeared to be overly optimistic expectations by the public for novel medications, particularly in chronic diseases with no cure. These authors and others suggest that this overestimation of efficacy may enable patients to meet their psychological needs such as hope, safety, a sense of control, action, and reassurance [55].

Callers to the MCC in our study were concerned about the potential effect of DMARDs on reproductive health; not only maternal but also paternal exposure to DMARDs before conceiving and during pregnancy. This is an area of concern for patients that is under-investigated in the literature. Many women of childbearing age with a chronic disease necessitating the use of DMARDs have difficulties finding adequate information related to pregnancy planning, pregnancy, and early parenting [56]. The effects of DMARDs on male reproduction is an even more neglected area, with sparse data on spermatogenesis and the effects on offspring [57]. Callers were worried about being in contact with other persons and animals, demonstrating patients’ perceptions of their medicines as potent and potentially toxic. This suggests that a greater effort to deliver targeted information in this domain is required.

Callers sought assistance to assess the benefit-risk balance of their DMARDs. This may, in part, be explained by the considerable mismatch between physicians’ and patients’ perspectives and preferences in the management of inflammatory arthropathies [58–61]. Others were interested in the availability, cost, and subsidised access to DMARDs - crucial information for patients with chronic conditions on relatively expensive medicines. The restrictions to limit access to subsidised treatments may add a layer of complexity to patients through their disease journey.

### Strengths and weaknesses

This is the first study to analyse real-life consumers’ information needs and concerns about DMARDs via a caller helpline. These enquiries were initiated proactively, predominately by the patients themselves or a significant other (family, friend, carer), for a genuine medicines information gap or concern related to a conventional or biologic DMARDs. Unlike other observational studies, where patients are invited to identify areas of concern or information needs through a questionnaire, interview or focus group, this study is a real-time capture of a population of DMARDs users. These callers had a genuine medicines information need unanswered in their usual physician-patient encounters or through other traditional sources where their uncertainty was sufficient to motivate them to actively help-seek. The longitudinal nature of the data captured over 8 years, including the timeframe when the use of bDMARDs became available, provide an understanding of how consumers might manage uncertainty associated with novel and expensive medicines.

This study has limitations, including that the MCC repository contains data collected as part of a health service which was not developed for research purposes. As such, variables of interest to future researchers may not be held in the database. DMARDs were extracted from the database using ATC codes; therefore, cases could not be consistently assessed by a specific DMARD indication, such as RA. Secondly, while the coding that emerged from the DMARD qualitative analysis was not validated by consumers, the coding framework and approach for MCC narrative analysis had previously been validated by consumer representatives and other key stakeholders on the MCC governance committee.

MCC callers represent only a part of the total group of DMARDs users in Australia. However, the representation of calls was comparable to ROCs, originating from all states and territories and across metropolitan, rural and remote areas. Following on from data completion in 2010, further DMARDs particularly bDMARDs and the targeted synthetic DMARDs have become available for use in Australia and data on these newer agents are therefore lacking [62, 63]. Furthermore, patients are more frequently accessing information regarding their medicines from various sources, predominantly online and through social media [64]. This increased exposure to information, as well as the inaccuracies and low readability of the content of many unreliable resources [65], may create different information gaps and concerns among patients not identified by our study.

## Conclusion

This study demonstrates that consumers have considerable uncertainty regarding DMARDs and they may overemphasise risk. Patients cautiously assess the benefits and risks of their DMARDs but when new treatments emerge, they tend to overestimate their effectiveness. Understanding the common themes driving medicines information-seeking related to DMARDs is key to addressing consumer information gaps and improving overall DMARD use and adherence.

## Supplementary information

**Additional file 1.**

## Data Availability

Restrictions apply to the public availability of the data that support the study findings. Data are however available from the authors upon reasonable request and with permission of the service funder, NPS MedicineWise (formerly National Prescribing Service, Australia).

## Abbreviations

ARIA: Accessibility/Remoteness Index of Australia
ATC: Anatomical Therapeutic Chemical
bDMARDs: Biologic disease-modifying antirheumatic drugs
cDMARDs: Conventional disease-modifying antirheumatic drugs
DMARDs: Disease-modifying antirheumatic drugs
MCC: Medicine call centre
NPS: National Prescribing Service
PBS: Pharmaceutical Benefit Scheme
ROC: Rest of calls
