# Supplementary figures and images for: Real-world questions and concerns about disease-modifying antirheumatic drugs (DMARDs): a retrospective analysis of questions to a medicine call center
Source: BMC Rheumatol. 2020 Jun 16;4:27. doi: 10.1186/s41927-020-00126-7 (PMC7296694; doi:10.1186/s41927-020-00126-7)

Additional information – Attachment 1:


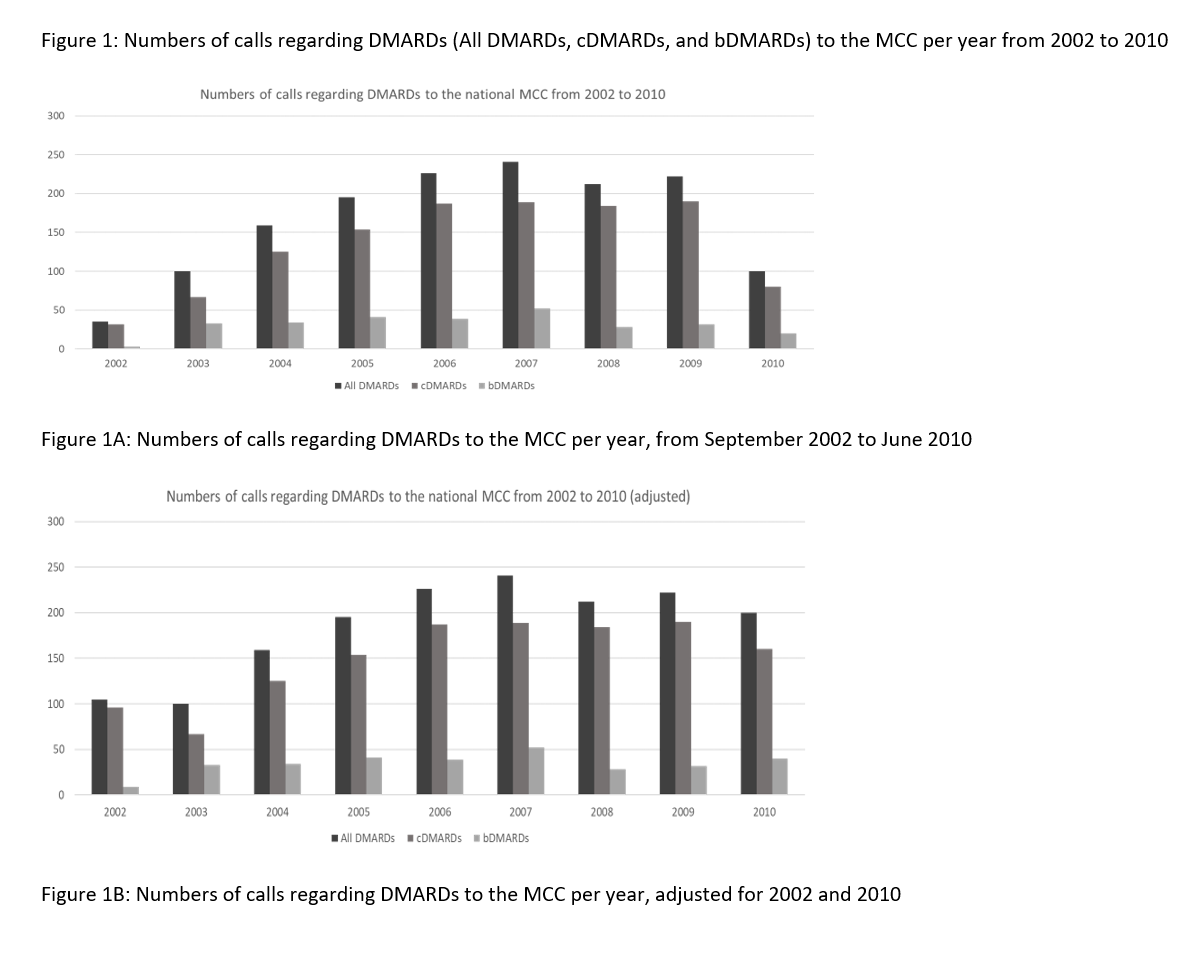

Supplement: Supplementary file 1 — Additional file 1. [file 41927_2020_126_MOESM1_ESM.docx]
